# Massive APOBEC3 Editing of Hepatitis B Viral DNA in Cirrhosis

**DOI:** 10.1371/journal.ppat.1000928

**Published:** 2010-05-27

**Authors:** Jean-Pierre Vartanian, Michel Henry, Agnès Marchio, Rodolphe Suspène, Marie-Ming Aynaud, Denise Guétard, Minerva Cervantes-Gonzalez, Carlo Battiston, Vincenzo Mazzaferro, Pascal Pineau, Anne Dejean, Simon Wain-Hobson

**Affiliations:** 1 Molecular Retrovirology Unit, Institut Pasteur, Paris, France; 2 Nuclear Organization and Oncogenesis Unit, INSERM U579, Institut Pasteur, Paris, France; 3 G.I. Surgery and Liver Transplantation Unit, Istituto Nazionale Tumori IRCCS, Milano, Italy; University of Southern California, United States of America

## Abstract

DNA viruses, retroviruses and hepadnaviruses, such as hepatitis B virus (HBV), are vulnerable to genetic editing of single stranded DNA by host cell APOBEC3 (A3) cytidine deaminases. At least three *A3* genes are up regulated by interferon-α in human hepatocytes while ectopic expression of activation induced deaminase (AICDA), an A3 paralog, has been noted in a variety of chronic inflammatory syndromes including hepatitis C virus infection. Yet virtually all studies of HBV editing have confined themselves to analyses of virions from culture supernatants or serum where the frequency of edited genomes is generally low (≤10^−2^). We decided to look at the nature and frequency of HBV editing in cirrhotic samples taken during removal of a primary hepatocellular carcinoma. Forty-one cirrhotic tissue samples (10 alcoholic, 10 HBV^+^, 11 HBV^+^HCV^+^ and 10 HCV^+^) as well as 4 normal livers were studied. Compared to normal liver, 5/7 APOBEC3 genes were significantly up regulated in the order: HCV±HBV>HBV>alcoholic cirrhosis. *A3C* and *A3D* were up regulated for all groups while the interferon inducible *A3G* was over expressed in virus associated cirrhosis, as was *AICDA* in ∼50% of these HBV/HCV samples. While AICDA can indeed edit HBV DNA *ex vivo*, A3G is the dominant deaminase *in vivo* with up to 35% of HBV genomes being edited. Despite these highly deleterious mutant spectra, a small fraction of genomes survive and contribute to loss of HBeAg antigenemia and possibly HBsAg immune escape. In conclusion, the cytokine storm associated with chronic inflammatory responses to HBV and HCV clearly up regulates a number of *A3* genes with A3G clearly being a major restriction factor for HBV. Although the mutant spectrum resulting from A3 editing is highly deleterious, a very small part, notably the lightly edited genomes, might help the virus evolve and even escape immune responses.

## Author Summary

Retroviruses and hepadnaviruses such as hepatitis B virus (HBV) are vulnerable to mutation by host cell single stranded DNA cytidine deaminases. The result is hypermutated viral peppered with uracil residues. While there are potentially 11 such human enzymes, the major players belong to the 7 gene APOBEC3 cluster on chromosome 22, some of which can be activated by anti-viral interferons. We investigated the nature and frequency of HBV editing in 41 cirrhotic samples following surgical removal of primary hepatocellular carcinoma. Numerous APOBEC3 genes were activated in the decreasing order HCV±HBV>HBV>alcoholic cirrhosis. We observed that APOBEC3G was the dominant restricting factor *in vivo* with up to 35% of HBV edited genomes. Among the HBV mutants generated by APOBEC3 editing, we found a small fraction of lightly APOBEC3G edited genomes that can impact HBV replication *in vivo* and possibly immune escape.

## Introduction

The human genome harbours a group of 11 genes encoding cytidine deaminases, the majority having substrate specificity for single stranded DNA (ssDNA) [1], [2], [3], [4], [5], [6], [7], [8], [9], [10]. These include the prototypical enzyme APOBEC1 (A1) and activation induced deaminase (AICDA). The large seven gene *APOBEC3* cluster spans ∼150kb at ch22q13.1 [11]. Two additional genes, *APOBEC2* and *APOBEC4*, show homology to the above, although no editing activity has been described so far for either. Many of the human APOBEC3 (A3) enzymes can edit the cDNA of numerous retroviruses, retrovirus elements and hepadnaviruses in tissue culture experiments [3], [4], [6], [12], [13], [14], [15], [16], [17], [18], [19], [20], [21]. Yet *in vivo* only the lentiviruses, of which human immunodeficiency virus (HIV) is the most notorious, hepatitis B virus (HBV), human papillomaviruses (HPV) and TTV genomes have proven to be edited [21], [22], [23], [24], [25], [26], [27].

The outcome of cytidine deamination is oxidation of the C4 amino group yielding uridine - in short cytidine deamination is mutagenic. The degree of editing can be as little as a few bases per kilobase or up to 90% of all cytidine residues, APOBEC3A deamination of hepatitis B virus DNA in tissue culture being a case in point [16]. In virology, mutations are generally related to the plus strand. Hence, so called G→A hypermutants reflect cytidine deamination of (−) stand DNA while C→T hypermutants reflect editing of the viral (+) strand. Due to the extent of editing, hypermutation can be seen as part of an innate anti-viral response. This theme is echoed by the fact that some *APOBEC3* genes are up regulated by interferon-α and –γ in a wide variety of cells including primary human hepatocytes [28], [29], [30], [31], [32], [33], [34], [35].

While *AICDA* expression is chiefly expressed in germinal centre centroblasts [36], ectopic expression of human *AICDA* has been shown in at least four settings, all involving chronic inflammation – HCV associated chronic hepatitis, *Helicobacter pylori* associated stomach cancer, human colitis and chronic inflammatory bile duct disease [37], [38], [39], [40], [41]. Transgenic mice bearing the human *AICDA* or *A1* genes invariably induce cancers, the organ specificity being dependent on the promoter [42], [43], [44]. As there is a long historical link between chronic inflammation and cancer [45], [46], the ensemble suggested a link between aberrant *AICDA* expression and, by inference, expression of other human APOBEC genes and editing of the nuclear genome [41], [44], [47]. Cancer frequently emerges from a background of cellular dysplasia. For the liver, cirrhosis is seen as a polyclonal proliferative disease, a prodrome that generally precedes the HCC. Yet virtually all APOBEC editing studies of the HBV genome have confined themselves to analyses of virions from culture supernatants or serum where the frequency of edited genomes is generally low (≤10^−2^, [21], [25]). In view of the above observations, we decided to look at the nature and frequency of HBV editing in cirrhotic liver samples taken during removal of a primary HCC. Given the finding of ectopic *AICDA* expression in HCV associated chronic hepatitis [39], [48], we chose to work with cirrhotic tissue from HBV mono- and HBV plus HCV double infections.

It is shown here that while human AICDA can indeed hyperedit HBV genomes in an *in vitro* setting, editing by AICDA is a rare event *in vivo*. By contrast, up to 35% of HBV genomes are edited *in vivo* by several A3 enzymes including A3G indicating that human A3 deaminases represent major restriction factors for HBV replication. Yet through HBV editing, A3 deaminases generate a mutant spectrum upon which selection can occur. It is suggested that APOBEC3 editing may contribute towards loss of HBeAg antigenemia and immune escape.

## Results

### Up regulation of APOBEC deaminases in cirrhosis

A succinct description of the 41 cirrhotic samples is given in Table S1. Ten DNA samples from patients with alcoholic cirrhosis, 10 HBV^+^, 11 HBV^+^HCV^+^ and 10 from HCV^+^ patients were analysed. Complementary DNA was made to total RNA extracted in parallel with the DNA samples and hybridized to a custom made TaqMan PCR chip comprising all 11 human cytidine deaminases related genes (*A1*, *A2*, *A3A–H*, *A4* and *AICDA*), a number of pro-inflammatory, apoptotic and mismatch repair genes as well as 3 reference genes (TRIM44, HMBS and LMF2) that have been validated for analyses of liver tissue.

**Figure 1:**
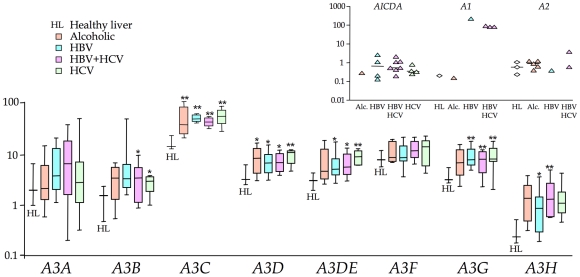
Transcription profiling of all 11 human cytidine deaminases in cirrhosis. The *A3* transcription data are in the form of box-whisker plots with the mean, quartiles, maxima and minima. Data are normalized to the expression levels of three invariable reference genes (TRIM44, HMBS and LMF2). Asterisks indicate statistically significant up regulation: ** 0.01<p<0.001; * 0.05<p<0.01. Inset) *AICDA*, *APOBEC1* and *APOBEC2* transcripts were detected in 0, 1 and 1 HL samples respectively but present in several HBV±HCV samples. *APOBEC4* transcripts were undetectable in all samples tested.

Compared to the 4 normal livers, there was significant up regulation of 2–5 *A3* genes in the order: HCV±HBV>HBV>alcoholic cirrhosis. *A3C* and *A3D* were up regulated for all groups, while the interferon inducible *A3G* was over expressed in virus associated, as opposed to alcoholic cirrhosis (Figure 1, Figure S1 & Table S2). While there was a trend towards increased A3A and A3F expression in all four groups, it never reached statistical significance. *AICDA* was up regulated in 15/31 HBV and/or HCV samples, indicating that ectopic expression of *AICDA* is also a feature of HBV liver disease (Figure 1 inset). By contrast expression was undetectable in the normal liver, which is why the data are not normalized as per *A3* data (Figure S1). *APOBEC1* was phenomenally expressed in one sample (#146) yet again normalization wasn't possible as it was weakly expressed in just one normal liver sample (Figure 1 inset). *APOBEC2* transcripts were relatively absent while *APOBEC4* was undetectable throughout. A number of cellular genes associated with inflammation were significantly up regulated, notably *FAS*, *FASLG*, *BCL2*, *IFNγ* and *LTA* (Table S2).

### AICDA can hyperedit HBV genomes *ex vivo*

**Figure 2:**
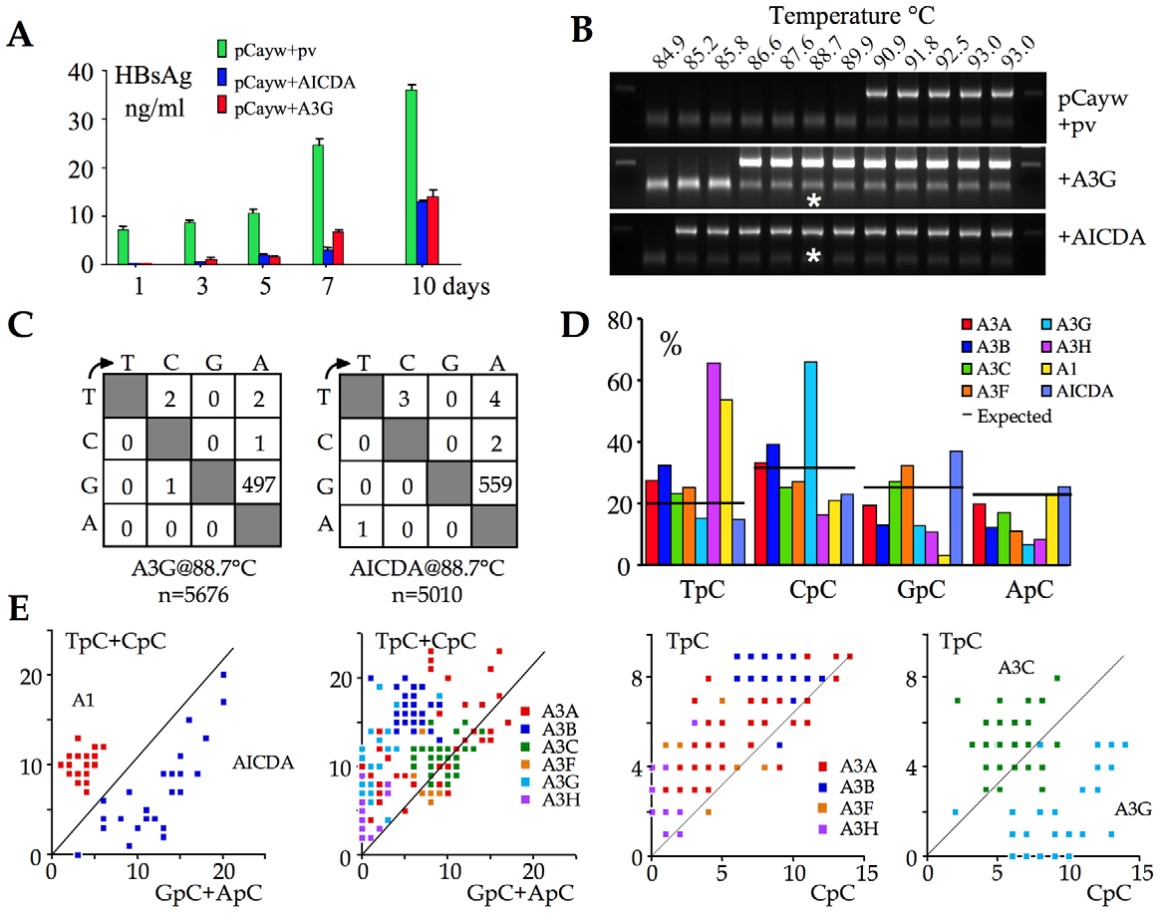
AICDA impacts HBV replication by hyperediting the genome. A) Macroscopic impact of AICDA and A3G on HBsAg secretion into the culture supernatant following transfection of the infectious molecular clone, pCayw. B) 3DPCR recovered A3G- and AICDA-edited HBV genomes down to 86.6°C and 85.2°C respectively. pv: empty plasmid vector and HBV alone. Asterisks refer to the samples cloned and sequenced. C) Mutation matrices for hyperedited X gene sequences derived from cloned 88.7°C 3DPCR products. n indicates the number of bases sequenced. D) Bulk dinucleotide context of HBV X region minus strand DNA by eight human cytidine deaminases. E) Clonal analysis of editing for individual edited sequences. The number of TpC+CpC vs. GpC+ApC targets edited per sequence are computed and represented on the y and x axes respectively. As the A3C and A3G genes were strongly up regulated (Figure 1) they have been separated from the others. Clonal analysis using TpC vs. CpC allows clear isolation of A3G from other A3 enzymes.

An infectious molecular clone of HBV was transfected into the hepatocyte derived Huh7 cell line along with human AICDA construct and A3G as positive control. HBsAg secretion in the supernatant was used as readout for editing at a macroscopic level. Figure 2A shows that both AICDA and A3G expression reduced HBsAg to levels ≤50% of control. Given that transfection frequencies were ∼30–40%, this suggests that the majority of genomes were edited. At 72 hours, total DNA was recovered from the supernatants and 3DPCR performed on a segment of the X gene [16], [21], [49], [50]. 3DPCR products were recovered as low as 85.2°C for the AICDA cotransfection compared to 86.6°C for the A3G control (Figure 2B). The 88.7°C 3DPCR products were cloned and sequenced. As can be seen from the mutation matrices (Figure 2C), AICDA (mean cytidine deamination frequency, *f_c_* = 42%, range 16–61%) was just as good as A3G at hyperediting HBV DNA (*f_c_* = 33%, range 7–98%).

As expected from extant data, AICDA editing of the HBV target was concentrated in GpC and ApC sites [1], [51] unlike its A3 counterparts (Figure 2D). Combined with previous data we now have a reference set for the HBV X region edited by 8/11 human cytidine deaminases [16]. Four deaminases (A3G, A3H, A1 and AICDA) showed polarized editing biases (Figure 2D) that can be used as hallmarks for specific deaminase activity *in vivo*. The singularity of AICDA and A3G editing with respect to other A3 enzymes becomes apparent when plotting the number of edited cytidine residues in selected dinucleotide contexts for single sequences (Figures 2E).

### A3G and not AICDA is the major restriction factor *in vivo*

**Figure 3:**
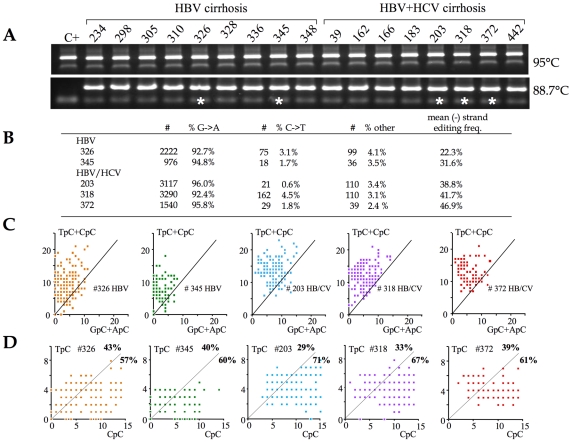
A3 deaminases are the major editors of HBV DNA *in vivo*. A) PCR and 3DPCR amplification at the normal and restrictive temperatures of 95°C and 88.7°C respectively. Sample codes are given above. Five 3DPCR samples identified by asterisks were chosen for cloning and sequencing. B) Summary of the unique hyperedited sequences in the form of the number and fraction of G→A (minus stand) edits, C→T (plus strand) and all other point mutations. The excess of GC→AT transitions over all other mutations varied from 23–40 fold. C) Clonal analysis using the number of TpC+CpC vs. GpC+ApC targets edited. The vast majority of patient sequences map to the area typical of APOBEC deaminases. D) Clonal analysis using the number of TpC vs. CpC targets edited. The majority of sequences map to the area typical of APOBEC3G (between 57–71% marked in bold face).

With these metrics in hand, 3DPCR was used to identify hyperedited HBV genomes from the cirrhotic samples. First round PCR DNA was performed on ∼0.5µg of total DNA using the X gene specific primers. In a second round, 3DPCR was performed at the restricting temperature of 88.7°C (Figure 3A). Fifteen of 17 DNA samples (88%) yielded robust amplification at the restrictive temperature. Five DNA samples – 2 HBV and 3 HBV+HCV - indicated by asterisks were cloned and sequenced. Hyperediting of the HBV genome was essentially confined to the minus DNA strand with only a handful of plus strand hypermutants, a finding in keeping with previous reports [21]. The mean cytidine editing frequencies were somewhat higher in the case of HCV co-infections (*f_c_* = 38.8%, 41.7% and 46.9%) compared to HBV monoinfection (*f_c_* = 23.3% and 31.6%, Figure 3B).

The dinucleotide editing context for these hyperedited genomes was remarkably uniform with a strong preference for CpC and an aversion for ApC, which fits rather well with the profile for A3G (not shown). However, it is possible that such averaging could mask the occasional AICDA edited genome. Accordingly, a clonal analysis was used to highlight editing by distinct enzymes (Figure 3C). Very few patient sequences mapped to the area characteristic of AICDA (Figure 3C. vs. Figure 2E), indicating that it is not a major editor *in vivo*. By contrast, between 57–71% of patient sequences fell within the area covered by A3G (Figure 3D vs. Figure 2E). The remaining sequences mapped to regions where there was considerable overlap between A3 deaminases (Figure 2E). As *A3C* was significantly up regulated in these liver samples and expressed at greater levels than any other *A3* gene (Figure 1), editing by A3C alone could explain the remainder.

**Figure 4:**
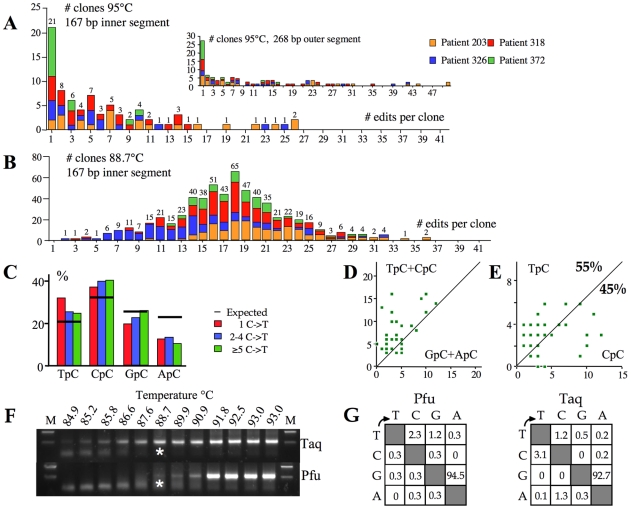
High frequency of A3 editing recovered by standard PCR. A) Frequency analysis of edited genomes as a function of the number of edits per sequence for 95°C derived PCR products. The 268 bp sequences were derived from the first round product. The sequences in insert to Figure 4A were stripped down to the size of the inner 167 bp locus and reanalyzed to allow comparison with the 3DPCR products. The numbers above the columns indicate the combined numbers of sequences across the four samples. B) Frequency distribution of edited sequences for the 3DPCR products obtained at 88.7°C. In order to calculate the bias resulting from PCR close to the denaturation temperature, let's assume that the frequency of clones with 1–17 edited cytidines reflected sub optimal amplification, while the profile in Figure 4A is close to the true distribution. By summing the number of clones with 1–17 and ≥18 edits for Figures 4A and 4B the estimated number with between 1–17 edits in Figure 4B is n, where 79/n = 7/300; n = 3386. As the number of clones in Figure 4B with 1–17 edits is 284, 3DPCR underestimates the true frequency by a factor of 3386/284, or ∼12. C) Bulk dinucleotide analysis of the 95°C sequences harbouring 1, 2–4 and ≥5 G→A transitions reveals the CpC hallmark of A3G editing. D & E) Clonal analysis reveals that editing was due to an APOBEC deaminase, approximately half being due to A3G. The smaller number of sequences used (n = 40) in these figures means that the values of 55% and 45% are less robust than for Figure 3C & 3D. F) 3DPCR amplification across a 85–93°C gradient using either Taq or Pfu DNA polymerase, the latter fails to amplify DNA containing dU, the product of A3 deamination. Asterisks indicate the PCR products cloned and sequenced. G) Mutation matrices of Pfu and Taq amplified HBV hypermutants given as percentages.

In order to determine accurately the overall hyperediting frequencies *in vivo*, we performed deep sequencing on cloned first round PCR DNA (95°C). To our surprise for 4/5 samples a sizeable proportion (10–35%) of X gene segments showed unmistakable signs of hyperediting. The frequency distribution of editing is shown in Figure 4A. Some sequences harboured up to 50/58 (86%) edited cytidines (Figure 4A inset), comparable to those recovered from a transfection experiment. Figure 4B shows the frequency distribution of hyperedited genomes from patients recovered by 3DPCR. That the two frequency distributions are distinct reflects selection against lightly edited genomes during 3DPCR, as previously noted [21]. For the 95°C derived sequences, dinucleotide and clonal analysis indicated that the edited bases showed the same hallmarks as those recovered by 3DPCR, that is a penchant for CpC typical of A3G editing with at least one other A3 enzyme explaining the remainder (Figure 4C–E). Hence, Figure 4B represents a subset of a more general distribution typified by Figure 4A.

Assuming that the highly edited part of the distribution (n≥18, Figure 4B) is unaffected by suboptimal amplification, then the expected number of edited genomes can be calculated to be 300×79/7 = 3386 (see legend to Figure 4). In other words ∼12 fold more genomes are edited than suggested by 3DPCR. Yet the actual number is probably higher as the X gene segment analyzed represents only ∼5% of the HBV genome. A genome with a wild type X gene sequence could be edited elsewhere. Hence, the real frequencies of slightly edited genomes are almost certainly higher.

### A subset of A3 edited genomes can be repaired

A sizeable proportion of neo-synthesized HBV DNA returns to the nucleus to augment the pool of cccDNA, the viral replication template. DNA bearing multiple dU residues might be degraded by the UNG-APE1 pathway or, if copied on the opposite strand, the resulting dU(−)∶dA(+) base pair might be corrected to dT∶dA. To explore this issue we performed first round and 3DPCR on sample #326 using Pfu polymerase. Like all archaeal DNA polymerases, Pfu is unable to amplify DNA templates bearing dU [52]. As can be seen, Pfu amplification recovered much less hyperedited DNA than Taq polymerase (Figure 4F). There were enough 3DPCR products from the 88.7°C amplification to allow cloning and sequencing. The genomes were exclusively edited on the minus strand (mean *f_c_* = 25%, Figure 4G) indicating that multiple dU∶dA pairs can be repaired to dT∶dA. The corresponding amplification using Taq (Figure 4G) yielded a comparable frequency (*f_c_* = 32%). These observations indicate that substantial repair does occur for a small number of hyperedited genomes.

### Precore/core mutations

The HBV core orf allows translation from the first, suboptimal AUG giving rise to the HBeAg precursor. Serum HBeAg is a marker for high viremia. Initiation from the second, optimal AUG, leads to synthesis of the capsid monomer, HBcAg. Over the course of a chronic infection, G→A mutations arise in the precore region, particularly at residue G1896 and to a lesser extent G1897 resulting in the loss of HBeAg synthesis and HBeAg seroconversion [53], [54]. These mutations result in a W28Stop change. There is no consensus as to whether this is due to A3 editing [20], [25], [55] despite the fact that G→A transitions in a run of four Gs (4Cs on the edited minus strand) are reminiscent of a hot spot for A3 deamination [21].

**Figure 5:**
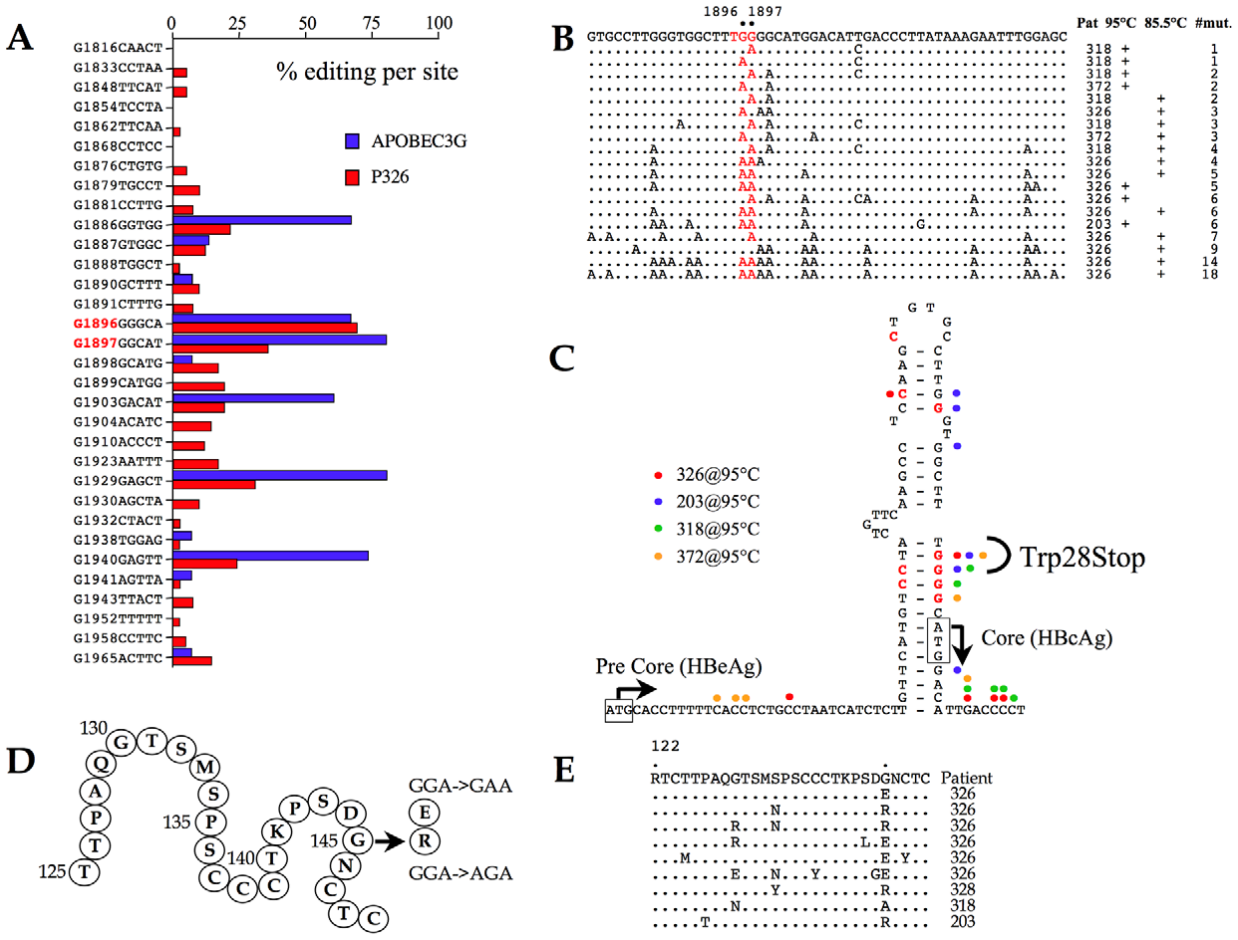
A3 editing of the precore and HBsAg coding regions. A) Site specific frequency analysis of A3G editing in the precore region from a tissue culture transfection experiment and from patient #326 recovered by 3DPCR at the restrictive temperature of 85.5°C. B) A selection of edited precore sequences *in vivo*. C) Correlation of G→A and C→T edited sites observed from the 95°C amplifications of samples #203, #318, #326 and #372 with those described from other studies with respect to the RNA stem loop structure implicit to DNA replication. D) Secondary structure of the common “a” determinant of HBsAg. The frequent G145R substitution and the G145R variant are noted along with the corresponding codon changes. E) A selection of A3 edited S region sequences bearing mutations in the G145 codon (standard PCR (95°C)). Such sequences represent <10% of the total.

To explore this issue, a region spanning the precore region was analyzed by PCR and 3DPCR from a HBV/A3G transfection and patient #326. As can be seen from Figure 5A, G1896 was indeed a hot spot for A3 editing (70–80% editing on the minus strand) both *in vivo* and for the A3G transfection experiment. Editing of the plus strand was also evident. This led us to analyze the core region for samples #203, #318 and #372 at 85.5°C (3DPCR) and 95°C (PCR). The data are summarized in Figure 5B. A gradient of editing is apparent with G1896 and G1897 apparently hot spots for A3 editing. Focussing on mutations identified at 95°C, which can be considered as relatively high frequency events compared to those recovered by 3DPCR, they overlap nicely with those reported in a number of previous studies (Figure 5C) suggesting that A3 editing may well explain their origin [54], [55].

### HBsAg mutants

The major viral surface antigen, HBsAg, is the basis of the highly successful subunit vaccine. Vaccine escape mutants are known, with a glycine to arginine (G145R) being the most frequent substitution mapping to the common double loop “a” determinant (residues 110–149, Figure 5D) [56], [57]. A G145E substitution is also known, as are a few other loop changes. The G145R mutant also emerges among HBV-immunoglobulin (HBIg) treated chronic carriers [58], [59] and goes undetected by standard diagnostic kits [60]. The local dinucleotide context associated with the G145 substitutions (Figure 5D) are again reminiscent of A3 editing. DNA spanning the “a” determinant was analyzed using the two-step PCR/3DPCR approach. Not surprisingly 3DPCR recovered hypermutants from all 4 samples tested at a restricting temperature of 85.5°C. The G145 codon was not a hotspot *in vivo*. As expected, the degree of editing was lower for genomes recovered at 95°C. Among sequences harbouring substitutions in codon 145 most were hypermutants with the G145E substitution while the G145R substitution was accompanied by other mutations (Figure 5E).

## Discussion

The transcriptome data shows that numerous *A3* genes are up regulated in virus associated cirrhosis, notably *A3B*, *A3C*, *A3G*, *A3H* and *AICDA* accompanied by a number of genes frequently up regulated in inflammatory tissues. By contrast, for alcoholic cirrhosis only two *A3* genes (*A3C* and *A3D*) were significantly up regulated. Using the dinucleotide context as a hallmark to identify *a posteriori* the deaminase *in vivo*, A3G clearly emerges as a major restriction factor for HBV replication in cirrhosis along with at least one other A3 deaminase, which could be A3C (Figure 3C, D). The degree of editing is every bit as extensive as in co-transfection experiments using the powerful CMV-IE promoter in tissue culture [16], [21]. The up regulation of *AICDA* doesn't impact HBV editing *in vivo* despite the fact that AICDA shows itself to be a potent editor of replicating HBV genomes (Figure 2). This apparent dichotomy could be explained by the neogenesis of lymphoid follicles harbouring AICDA^+^ centroblasts, a feature associated with chronic inflammation [61], [62], [63], or circulating AICDA^+^ B cells as has been described for chronic HCV infection [64], [65], [66].

That A3G is the dominant A3 enzyme fits nicely with the fact that of all the *A3* genes, *A3G* is the most strongly up regulated by interferon-α in primary hepatocytes [29]. Other reports show that the gene is also sensitive to induction by interferon–γ [28], [30], [34]. Pegylated interferon–α is used to treat a proportion of HBV infected individuals and the present data may explain part of that effect [67]. A recent report made a link between IFN-α treatment and HBeAg seroconversion, although they concluded that the link was tenuous given the low levels of editing in sera [25]. Although they didn't sequence precore DNA they used a 3DPCR approach. As shown here, the technique tends to underestimate levels by ∼10 fold (Figure 4). Accordingly hyperedited mutant frequencies reported in that study [25] can be revised upwards to ∼2–33%, in excellent agreement with the present findings (<2–35%). Given the strong impact of A3 deaminases on HBV replication in late stage disease, where does the virus replicate, especially as it is not known to encode an IFN or A3 antagonist? Some simple possibilities could be interferon resistant or A3^low^ hepatocytes.

On a background of HBV+HCV double infection, HBV titres are generally lower [68], [69], as though HCV infection rendered the liver an even more hostile environment for HBV. It might be that the strong pro-inflammatory responses associated with HCV immune responses induce *A3* genes with their detrimental effect on HBV. That the mean HBV cytidine deamination frequency was higher from the double infection compared to the monoinfection is the result expected if this hypothesis is correct (Figure 3B). As there has been debate as to the importance of *A3G* alleles in HIV disease (H186R [70]), it is possible that polymorphisms in the *A3G* gene impact the outcome of HBV infection. Certainly the most striking of all *A3* polymorphisms, Δ*A3B^−/−^*, doesn't impact HBV disease [22], [71].

Given their ability to hypermutate DNA, A3 enzymes generate complex mutant spectra, the vast majority probably being deleterious. Even so, lightly A3 edited genomes predominated (Figure 4A) while a small fraction resists degradation and are repaired to standard DNA as the Pfu/Taq comparison shows (Figure 4F). Thereafter selection will operate on the remaining genomes. It would seem that IFN induced A3 editing may indeed lead to the occasional emergence of variants, of which the precore C1896T and/or C1897T and HBsAg G145R,E mutants are tangible examples. In this respect, there are remarkable parallels between some RNA viruses and IFN-α induction of the dsRNA adenosine deaminase, ADAR-1L. Editing by this enzyme of A1012 in the hepatitis D virus genome is essential to complete replication [72], [73]. Similarly, a handful of edited adenosine residues allows escape of respiratory syncytial viruses from monoclonal antibodies [74], [75].

In conclusion, the cytokine storm associated with chronic inflammatory responses to HBV and HCV clearly up regulates a number of A3 genes with A3G clearly being a major restriction factor for HBV. Could this also be a feature of other chronic inflammatory syndromes or even autoimmune diseases? Although the mutant spectrum resulting from A3 editing is highly deleterious, a very small part, notably the lightly edited genomes might help the virus evolve and even escape immune responses.

## Materials and Methods

### Patients, samples, RNA extraction, integrity and amplification

Patients were predominantly males, the mean ages being 60 years (HBV), 63 years (HBV+HCV), 64 years (HCV) and 67 years (alcoholic). All were negative for HIV. No patient was on interferon therapy in the months prior surgery. The study was approved by an Institutional Human Research Review Board (RBM 2005–019) and by Institut Pasteur. Written informed consent was obtained for each patient.

Forty-one cirrhotic samples as well as 4 normal livers were dissected and directly frozen in liquid nitrogen after surgical removal. Healthy samples represent tissue surrounding benign tumours such as angioma or focal nodular hyperplasia. Total RNA extraction was performed by a phenol-based method (Euromedex, Souffelweyersheim, France). DNase treatment was performed on 10 µg of total RNA using a DNA-free kit (Ambion). The RNA concentration and integrity were assessed using 100 ng of each RNA isolate to perform a capillary gel electrophoresis analysis (RNA 6000 Nano chip kit, Agilent Technologies, Palo Alto, CA) to establish an RNA integrity index (RIN). All the samples had acceptable quality, 86% with 7.0<RIN<10.0 and 14%, 4.1<RIN<6.9. Reverse transcription of 1 µg RNA was performed in a final volume of 20 µl (High-Capacity cDNA Archive kit, Applied Biosystems).

### Real time quantitative PCR based gene expression

Messenger RNA were quantified by the TaqMan Low Density Array (TLDA) technology (Applied Biosystems, Courtaboeuf, France). Pre-designed hydrolysis probe and primer sets for target genes were factory loaded into the 384 wells of TLDA configured to contain duplicates per target gene (Table S2). Quantitative PCR was performed using cDNA samples corresponding to 400 ng of starting RNA and TaqMan Universal PCR Master Mix (Applied Biosystems) for 48 target genes in duplicate. QPCR conditions were one step of 94.5°C for 10 min. followed by 40 cycles at 97°C for 30 sec. and 59.7°C for 1 min. on a 7900HT Micro Fluidic Card instrument (Applied Biosystems).

For data analysis, gene expression values were determined using the calculation of the relative quantitation (RQ) of target genes normalized to a calibrator corresponding to 4 normal livers. RQ calculation was performed using the {Delta}{Delta}CT method using the geometric mean of three reference genes (TRIM44, HMBS and LMF2). The three references genes were selected among 12 constant genes arising from a previous array analysis of 70 HCC samples and 9 normal livers for which we applied the algorithms described [76] in the geNorm manual available on the web site http://medgen.ugent.be/~jvdesomp/genorm The study was performed according to the Minimum Information for Publication of Quantitative Real-Time PCR Experiments (MIQE) and redaction of the manuscript according to the RDML (Real-Time PCR Data Markup Language) data standard (http://www.rdml.org).

### Molecular biology

The pCayw plasmid and all *APOBEC* and *AICDA* expression plasmids have been previously described as have the cell lines and transfection protocols [16], [21]. Transfections were performed independently in triplicate on Huh7 cells and supernatant HBsAg was measured every day with the Monalisa HBsAg PLUS Kit (Bio-Rad). 3DPCR [50] was performed on a Eppendorf gradient Mastercycler S programmed to generate a 4–12°C gradient in the denaturation temperature. A fragment of the X region was amplified by employing a nested procedure. The first-round primers were: 5′Xout: 5′CGCAAATATACATCGTATCCAT and 3′Xout: AAGAGTYYTYTTATGTAAGACYTT, where Y is T,C and R is A,G. First PCR, conditions were: 5 min 95°C then (30 sec, 95°C; 30 sec, 60°C; 1 min, 72°C) ×35. Nested PCR was performed with 1/50 of the first round, primers were: 5′Xin: ATGGCTGCTARGCTGTGCTGCCAA and 3′Xin: AAGTGCACACGGTYYGGCAGAT, amplification conditions were: 5 min (82–93°C), then (1 min, 82–93°C; 30 sec, 60°C; 1 min, 72°C) ×35 then at 10 min 72°C.

The precore amplification was performed as following, first PCR, conditions were: 5 min 95°C then (30 sec, 95°C; 30 sec, 55°C; 1 min, 72°C) ×35 with primers 5′PreCout: GTACTAGGAGGCTGTAGGCATA and 3′PreCout: 5′ AGAGCTGAGGCGGTATCTAGAA. Nested PCR was performed with 1/50 of the first round, conditions were: 5 min (82–93°C), then (1 min, 82–93°C; 30 sec, 55°C; 1 min, 72°C) ×35, then 10 min at 72°C and primers were: 5′PreCin: TAAATTGGTCTGCGCACCAGCA and 3′PreCin: GATCTCGTACTGAAGGAAAGAA.

Amplification of the HBsAg was performed with a nested procedure, first PCR conditions were: 5 min 95°C then (30 sec, 95°C; 30 sec, 60°C; 1 min, 72°C) ×35, primers were: 5′HBsout: CGGCGTTTTATCATCTTCCTCTTCAT and 3′HBsout: CATCCATATAACTGAAAGCCAAACAGT. Nested PCR was performed with 1/50 of the first round, conditions were 5 min (82–93°C), then (1 min, 82–93°C; 30 sec, 60°C; 1 min, 72°C) ×35 then 10 min at 72°C with primers, 5′HBsin: TCTTCATCCTGCTGCTATGCCTCAT and 3′HBsin: AAAGCCCTACGAACCACTGAACAAAT.

PCR and 3DPCR products were purified from agarose gels (Qiaex II kit, Qiagen, France) and ligated into the TOPO TA cloning vector (Invitrogen, France). The 88.7°C 3DPCR products obtained from X gene amplification were chosen as experience shows they provide a wide range of edited HBV genomes. While products analyzed at lower temperatures are more edited, they proved to be more homogeneous. Sequencing was outsourced to Cogenics. All mutations were verified on the chromatogram.

## Supporting Information

Figure S1 APOBEC3 transcriptome data normalized to mean values for the four normal liver samples. Asterisks indicate statistically significant up regulation: ** 0.01<p<0.001; * 0.05<p<0.01.
(0.38 MB TIF)

Table S1 Brief description of the patients and genotype of the accompanying tumour. CTNNB1 = β-catenin gene.
(0.74 MB TIF)

Table S2 Statistically significant p values for gene up regulation in cirrhotic tissue from four distinct groups compared to 4 healthy liver controls. NS - not significant, p>0.05.
(0.82 MB TIF)
