# Supplementary figures and images for: Massive APOBEC3 Editing of Hepatitis B Viral DNA in Cirrhosis
Source: PLoS Pathog. 2010 May 27;6(5):e1000928. doi: 10.1371/journal.ppat.1000928 (PMC2877740; doi:10.1371/journal.ppat.1000928)

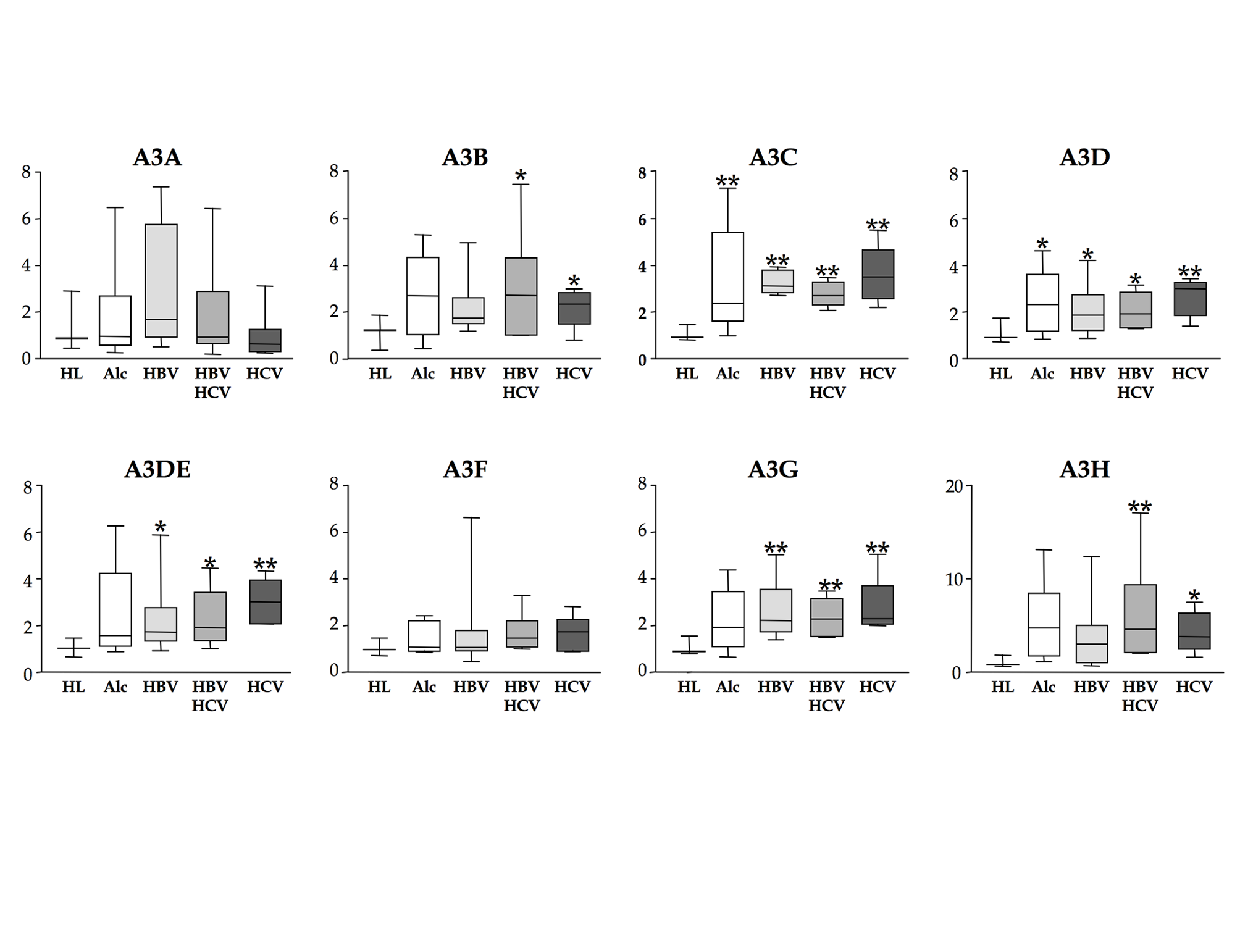

Supplement: Figure S1 — APOBEC3 transcriptome data normalized to mean values for the four normal liver samples. Asterisks indicate statistically significant up regulation: ** 0.01<p<0.001; * 0.05<p<0.01. (0.38 MB TIF) [file ppat.1000928.s001.tif]

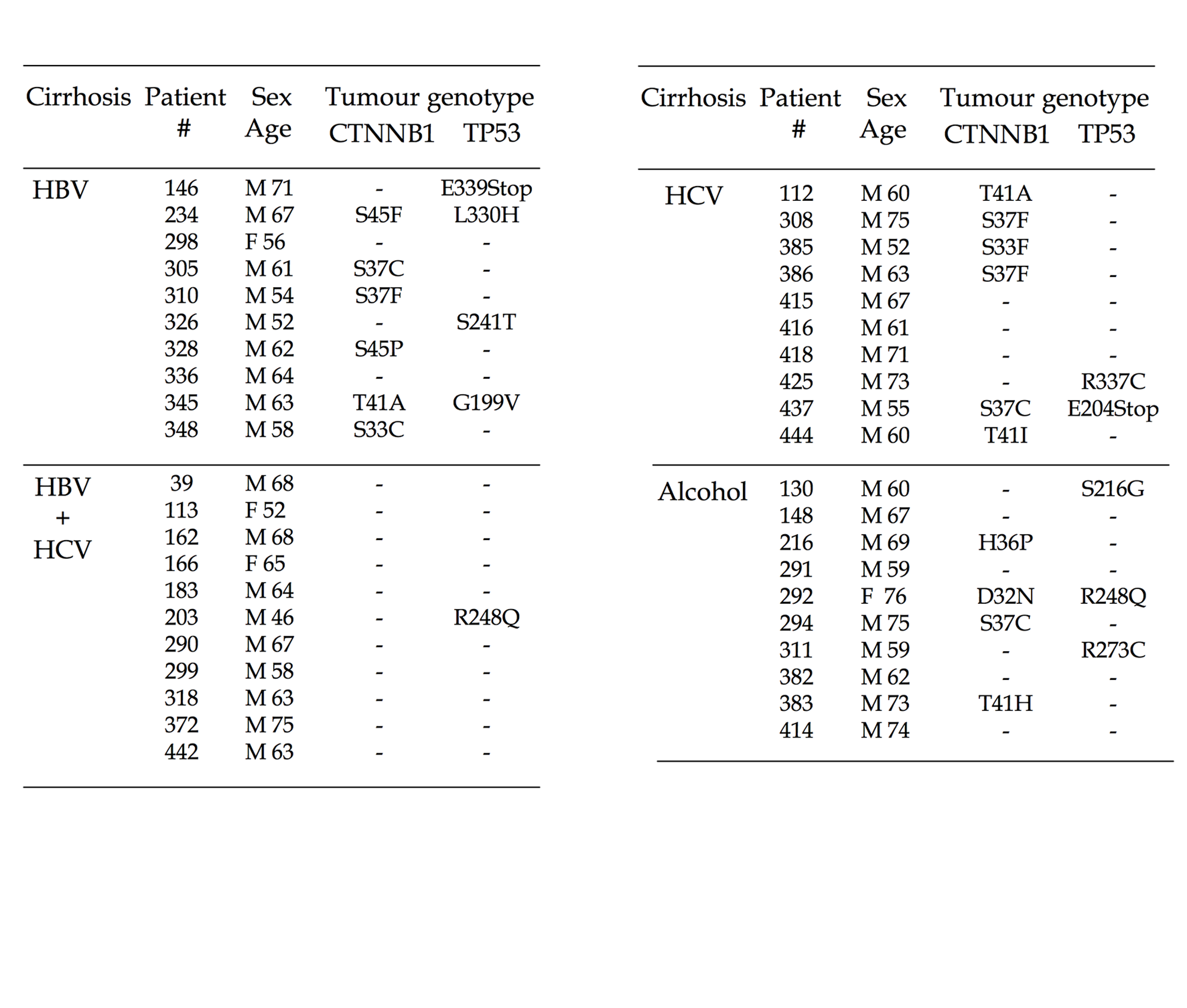

Supplement: Table S1 — Brief description of the patients and genotype of the accompanying tumour. CTNNB1 = β-catenin gene. (0.74 MB TIF) [file ppat.1000928.s002.tif]

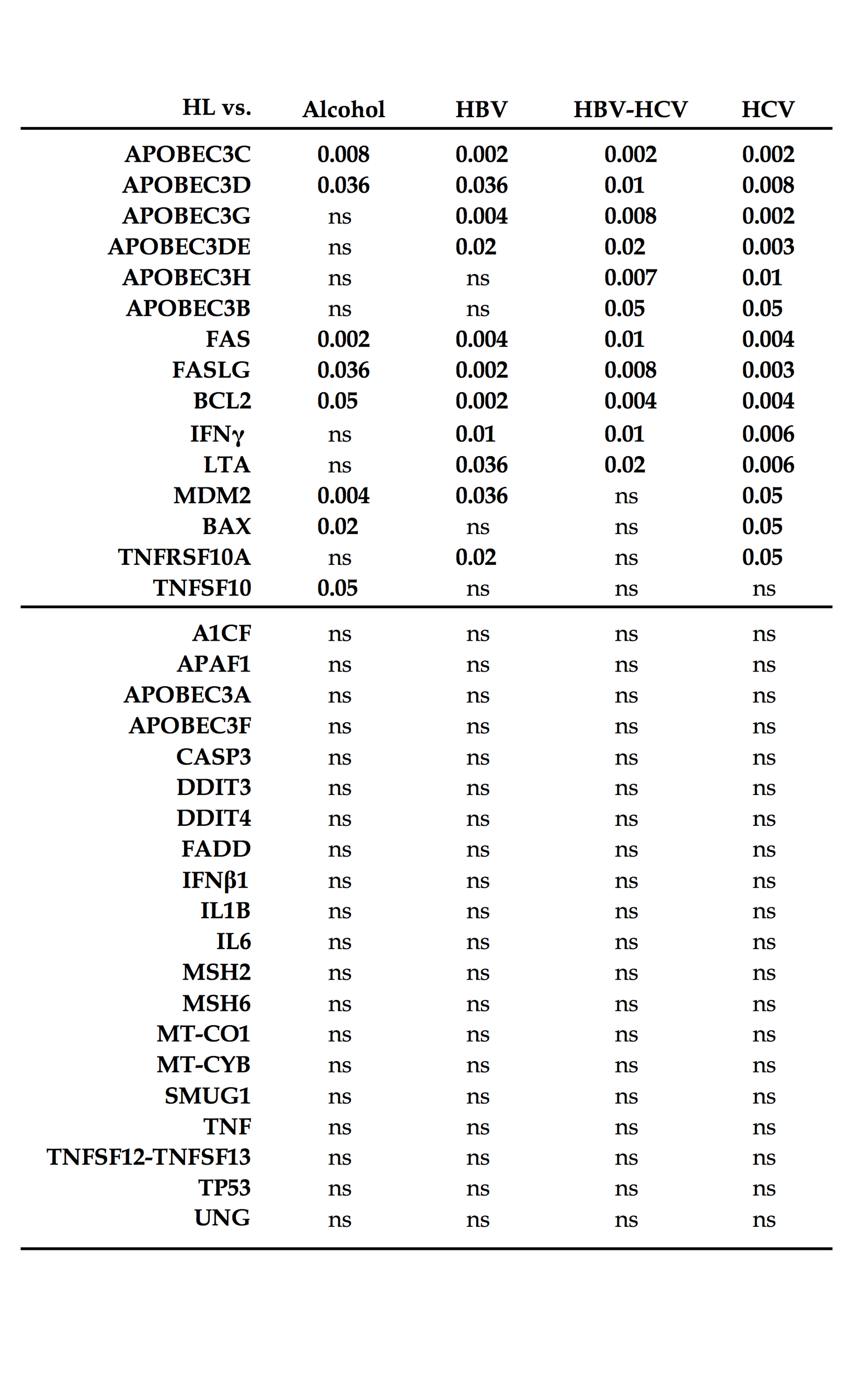

Supplement: Table S2 — Statistically significant p values for gene up regulation in cirrhotic tissue from four distinct groups compared to 4 healthy liver controls. NS - not significant, p>0.05. (0.82 MB TIF) [file ppat.1000928.s003.tif]
